# The Zebrafish Homologue of the Human DYT1 Dystonia Gene Is Widely Expressed in CNS Neurons but Non-Essential for Early Motor System Development

**DOI:** 10.1371/journal.pone.0045175

**Published:** 2012-09-28

**Authors:** Jonathan J. Sager, Gonzalo E. Torres, Edward A. Burton

**Affiliations:** 1 Department of Neurobiology, University of Pittsburgh School of Medicine, Pittsburgh, Pennsylvania, United States of America; 2 Department of Neurology, University of Pittsburgh School of Medicine, Pittsburgh, Pennsylvania, United States of America; 3 Department of Pharmacology, University of Pittsburgh School of Medicine, Pittsburgh, Pennsylvania, United States of America; 4 Department of Microbiology and Molecular Genetics, University of Pittsburgh School of Medicine, Pittsburgh, Pennsylvania, United States of America; 5 Pittsburgh Institute of Neurodegenerative Diseases, University of Pittsburgh School of Medicine, Pittsburgh, Pennsylvania, United States of America; 6 Geriatric Research, Education and Clinical Center, Pittsburgh Veterans Affairs Healthcare System, Pittsburgh, Pennsylvania, United States of America; 7 Department of Neurology, Pittsburgh Veterans Affairs Healthcare System, Pittsburgh, Pennsylvania, United States of America; University of Utah School of Medicine, United States of America

## Abstract

DYT1 dystonia is caused by mutation of the TOR1A gene, resulting in the loss of a single glutamic acid residue near the carboxyl terminal of TorsinA. The neuronal functions perturbed by TorsinA[ΔE] are a major unresolved issue in understanding the pathophysiology of dystonia, presenting a critical roadblock to developing effective treatments. We identified and characterized the zebrafish homologue of TOR1A, as a first step towards elucidating the functions of TorsinA in neurons, *in vivo*, using the genetically-manipulable zebrafish model. The zebrafish genome was found to contain a single alternatively-spliced *tor1* gene, derived from a common ancestral locus shared with the dual TOR1A and TOR1B paralogues found in tertrapods. *tor1* was expressed ubiquitously during early embryonic development and in multiple adult tissues, including the CNS. The 2.1 kb *tor1* mRNA encodes Torsin1, which is 59% identical and 78% homologous to human TorsinA. Torsin1 was expressed as major 45 kDa and minor 47 kDa glycoproteins, within the cytoplasm of neurons and neuropil throughout the CNS. Similar to previous findings relating to human TorsinA, mutations of the ATP hydrolysis domain of Torsin1 resulted in relocalization of the protein in cultured cells from the endoplasmic reticulum to the nuclear envelope. Zebrafish embryos lacking *tor1* during early development did not show impaired viability, overt morphological abnormalities, alterations in motor behavior, or developmental defects in the dopaminergic system. Torsin1 is thus non-essential for early development of the motor system, suggesting that important CNS functions may occur later in development, consistent with the critical time window in late childhood when dystonia symptoms usually emerge in DYT1 patients. The similarities between Torsin1 and human TorsinA in domain organization, expression pattern, and cellular localization suggest that the zebrafish will provide a useful model to understand the neuronal functions of Torsins *in vivo*.

## Introduction

Dystonia is characterized by sustained, patterned involuntary muscle contractions resulting in abnormal postures and twisting movements [1]. The most common form of hereditary dystonia is linked to the DYT1 locus; initial clinical manifestations of DYT1 dystonia usually occur during late childhood and adolescence [2]. DYT1 dystonia is caused by mutations in the TOR1A gene encoding TorsinA [3]. Nearly all patients harbor an in-frame trinucleotide GAG deletion in exon 5 of TOR1A, resulting in the loss of a single glutamic acid (ΔE) near the carboxyl terminal of TorsinA. The mutant allele gives rise to dystonia that is inherited as an autosomal dominant trait. Only approximately 40% of mutation carriers manifest dystonia [2]. The reasons for this reduced penetrance are not known; several hypotheses have been suggested, including modifying genes and environmental stressors [4].

TorsinA is a member of the AAA^+^ superfamily of proteins (ATPase associated with various cellular activities). Torsins are found only in metazoan animals, and a unique subset has arisen in the vertebrate lineage [4]. Members of the AAA^+^ family have roles in a diverse array of cellular functions [5]. *In vitro* studies have implicated human TorsinA in numerous cellular processes including cytoskeletal dynamics [6], synaptic vesicle cycling [7] and the secretory pathway [8], [9]. TorsinA is expressed in a wide variety of cell types [10] and colocalizes predominately with endoplasmic reticulum (ER) markers [11]. Mutant TorsinA[ΔE] shows aberrant cellular localization, being redistributed from the ER to the nuclear envelope (NE) in some cell lines [12], and forming cytoplasmic membranous whorls in others [11]. Similar to mutant TorsinA[ΔE], disruption of the Walker B ATP hydrolysis domain of TorsinA by mutagenesis also resulted in relocalization to the NE [9], [12]. Because comparable Walker B domain mutations in other AAA^+^ family members exhibit stabilization of substrate interactions [13], [14], the similar redistribution of TorsinA by ATP hydrolysis domain and ΔE mutations led to the hypothesis that both mutations prevent disengagement of TorsinA from a NE resident protein [15]. However, accumulating data suggest that the ATP hydrolysis domain and ΔE mutants may not be mechanistically equivalent; the two mutants differ in the formation of membranous whorls [15] and in the strength of co-immunoprecipitation with two putative NE substrates [16].

Although these studies have started to elucidate the cellular functions of Torsins, the mechanisms by which mutant TorsinA[ΔE] causes dystonia are not understood. Despite the dramatic clinical abnormalities, brain tissue from DYT1 dystonia patients is histopathologically unremarkable at autopsy, suggesting that aberrant activity or connectivity in neural circuits might underlie the pathophysiology of dystonia [17]. Consequently, there has been significant interest in generating model systems to gain insights into the functions of TorsinA in neurons and motor circuits *in vivo*. In *C. elegans*, mutations in the Torsin-related *ooc-5* gene disrupted spindle orientation and PAR protein polarity at the 2-cell stage of development, thereby preventing asymmetric divisions and cell fate determination [18]. The *D. melanogastor* genome contains a single Torsin family member, *dtorsin*. Knockdown of *dtorsin* in the retina by RNA interference altered the cellular organization of pigment granules, suggesting a role in intracellular transport [19]. Analysis of *dtorsin^−/−^ mutants* suggested that *dtorsin* may act as a positive regulator of GTP cyclohydrolase, an enzyme important in the production of BH4, a limiting cofactor in dopamine synthesis [20]. In mice, multiple strategies have been employed to generate a transgenic model of dystonia. Although these models have yielded insights into the neuronal mechanisms perturbed by expression of TorsinA[ΔE], none of these models exhibits clinical dystonia [21]–[27]. Inactivation of endogenous murine TOR1A by homologous recombination caused perinatal lethality, despite the absence of overt developmental morphological abnormalities. Transgenic mice overexpressing human TorsinA[ΔE] under the immediate-early promoter from cytomegalovirus (CMV) did not develop dystonia, although a deficit in motor learning was identified [25], as well as a reduced response to amphetamine [28] and changes in the bidirectional plasticity of glutamatergic synapses between cortical neurons and striatal medium spiny neurons [29]. Although these results confirm an important role for TorsinA in motor control, the current strategies have not resulted in a model that would allow physiological, pharmacological and molecular studies to understand the pathophysiology of dystonia and develop treatments for this condition.

There has been increasing interest in the use of zebrafish models to provide novel insights into mechanisms of human disease [30], and a zebrafish model of DYT1 dystonia could be very useful. Zebrafish can be manipulated to be transparent during larval development, allowing the deployment of fluorescent reporter proteins and chemical probes to monitor activity in neural circuits [31], dynamic changes in gene expression [32] and cellular movement and morphology *in vivo* [33]. Zebrafish can be genetically modified using simple and widely available methods, allowing hypotheses concerning molecular mechanisms to be tested rapidly. Since zebrafish produce large clutches of embryos, screens for genetic modifiers are feasible. In addition, the ability to house zebrafish larvae in 96-well plates facilitates exposure to chemical libraries, permitting discovery of small molecule modifiers and candidate novel therapeutic agents [34]. With specific relevance to dystonia models, the availability of automated neurobehavioral assays would allow high-throughput chemical or genetic screens to be driven by phenotypic abnormalities relevant to the function of motor circuits [35]. Finally, the zebrafish presents an accessible vertebrate model for neurophysiological studies to determine the effects of genetic manipulations on the function of motor systems [36], [37]. Zebrafish models have recently been used to study several other movement disorders, including Parkinson’s disease [38], [39], Huntington’s disease [40], and ataxias [41]. The high degree of phylogenetic conservation of genes and molecular pathways relevant to disease pathogenesis, and the many neurochemical and neuroanatomical similarities shared between vertebrates of different classes [42], [43], suggests that insights yielded by zebrafish models will be applicable to human disease.

As a first step towards generating a zebrafish model of DYT1 dystonia, we identified the zebrafish homolog of TorsinA. Here, we show that the zebrafish genome contains a single *tor1* gene, expressed as two splice variants, in contrast to the dual TOR1A and TOR1B paralogues found in tetrapod genomes. Zebrafish Torsin1 shares a similar protein domain structure with human TorsinA; like its human homologue mutations in the ATP hydrolysis domain of Torsin1 resulted in redistribution of the protein to the nuclear envelope. Similar to murine TOR1A, loss of *tor1* during early development did not cause morphological defects in the nervous system, loss of dopamine neurons or deficits in spontaneous movement. Taken together, our findings provide an essential basis for further studies to elucidate the functions of Torsin proteins in CNS function and for future development of a zebrafish DYT1 dystonia model.

## Methods

### Ethics Statement

All studies were carried out in accordance with NIH guidelines for animal care and use, and with approvals from the University of Pittsburgh Institutional Animal Care and Use Committee.

### Zebrafish

Adult strain AB zebrafish were maintained at 28.5°C and euthanized by deep tricaine anesthesia followed by exposure to ice-cold water. Embryos were raised in E3 buffer (5 mM NaCl, 0.17 mM KCl, 0.33 mM CaCl_2_, 0.33 mM MgSO_4_) supplemented, where necessary, with 0.003% 1-phenyl 2-thiourea (PTU) to inhibit pigmentation.

### RT-PCR and Rapid Amplification of cDNA Ends (RACE)

RT-PCR and RACE were carried out as previously described [44]–[46]. Briefly, total RNA was extracted from adult Zebrafish brain (*RNAqueous,* Ambion, Austin, TX) and reverse transcribed (*SuperScript III*, Invitrogen, Carlsbad, CA) using oligo-dT or 3′RACE adapter primers (*FirstChoice RACE*, Ambion, Austin, TX). For 5′RACE, RNA was treated with tobacco acid pyrophosphatase and RACE adapter was ligated prior to reverse transcription. Zebrafish *tor1* was amplified by RACE using adapter primers and gene-specific primers (5′ RACE: 5′-GGCTTTCAGGATGACTTGACCT-3′; 3′ RACE: 5′-TTCAGGTGGCTCTGGATTTC-3′) and by RT-PCR using a pair of gene-specific primers (*tv1F* = 5′-CGGAAGTGGGTCGTCATTAT-3′; *tv2F* = 5′-CCACCCTGTTTCCGACTAAA-3′; *ex5R*: 5′-CCCTTTAAAACAGGGACACG-3′). *bactin1* was amplified as a control (bactin1F: 5′-CCAACTGGGATGATATGGAGAAGA-3′; bactin1R: 5′-CAATGGTGATGACCTGTCCGTC-3′). PCR products were cloned in pGEM-T (Promega, Madison, WI) and sequenced. Expression vectors for Torsin1-GFP fusion proteins were generated by inserting the coding sequence of *tor1* in frame into pCS2^+^eGFP, resulting in the fusion of GFP to the C-terminus of Torsin1. Mutations were introduced into the coding sequence of the *Torsin1a-eGFP* fusion construct by site-directed mutagenesis (*QuikChange*, Stratagene, La Jolla, CA).

### Northern Blot

Northern blots were carried out as previously described [44], [46]. Briefly, total RNA, extracted from adult zebrafish brain (*RNAqueous*, Ambion, Austin, TX), was electrophoretically separated in a 1% agarose fomaldehyde/MOPS gel, and transferred to a Nytran-N membrane (Schleicher and Schuell BioScience, Keene, NH). Labeled *tor1* antisense probe was generated by *in vitro* transcription with UTP-digoxigenin (Roche, Indianapolis, IN). Membrane was hybridized in Ultra-Hyb (Ambion, Austin, TX) containing Torula RNA (1 mg/mL; Sigma, St. Louis, MO) and labeled cRNA probe (25 ng/mL) at 68°C. Stringency washes were carried out in 0.1× SSC/0.1% SDS at 68°C. Probe was detected using anti-DIG antibody conjugated to alkaline phosphatase (Roche, Indianapolis, IN) with a light-emitting AP substrate (*CDP-star*, Roche, Indianapolis, IN).

### Whole Mount RNA in situ Hybridization

*In situ* hybridization was carried out as previously described [44], [46]. Briefly, embryos were fixed in 4% paraformaldyde overnight at 4°C. After washing in PBS, embryos were dehydrated with methanol and stored at −20°C. Embryos were next washed in acetone at −20°C, hydrated with 50%, 30% methanol and then washed in PBS. Permeabilization with Protease-K (10 µg/µL) was followed by post-fixing in 4% PFA and washing in PBTw (PBS, 0.1% Tween, 0.2% BSA). Embryos were incubated in UltraHyb Buffer (Ambion) containing 1 mg/mL, Torula RNA, 50 µg/mL heparin and 150 ng/mL cRNA probe. Washes were carried out in 50% formamide, 2× SSC, 0.3% CHAPS, then 2× SSC, 0.3% CHAPS, followed by 0.2× SSC, 0.3% CHAPS. Blocking in 1× maleic acid buffer pH7.4, 1% blocking agent (Roche, Indianapolis, IN), was followed by incubation in alkaline phosphatase conjugated anti-DIG antibody at 37°C. After washing in PBS, antibody hybridization was detected with BM Purple (Roche, Indianapolis, IN).

### Torsin1 Antibody and Western Blots

Torsin1 antibody was generated by inoculating rabbits with the peptide CPDKEVVEKMAHD derived from the C-terminus of Torsin1, linked at its N terminus to keyhole limpet hemacyanin (New England Peptide). Crude antiserum was affinity purified against the peptide using a column (*SulfoLink*, Pierce, Rockford, IL) as previously described [47]. Transfected cells or adult zebrafish brains were sonicated in RIPA buffer (10 mM Tris, 150 mM NaCl, 1 mM EDTA, 0.1% SDS, 1% Triton X-100, and 1% sodium deoxycholate, pH7.4) and protein concentrations were measured using BCA assay. Equal amounts of protein were resolved on a 10% polyacrylamide gel, transferred to nitrocellulose, and blocked with 10% milk in TBS (50 mM Tris-HCl, 150 mM NaCl, 0.2% Tween-20). Torsin1 affinity-purified antibody was used at a concentration of 1∶1000, and was detected using HRP-conjugated proteinA (1∶4000; GE Healthcare, Piscataway, NJ). For peptide competition experiments, Torsin1 antibody was pre-incubated with 760 µM peptide in PBS for 2.5 hours at 37°C.

### Immunohistochemistry

Adult zebrafish brains were fixed in 4% PFA and cryoprotected in a sucrose series. 14 µm cryosections were incubated with Torsin1 antibody (1∶500) in carrier buffer (PBS, 1% goat serum, 1% BSA). Primary antibody was detected using an anti-rabbit HRP-conjugated antibody (1∶1000; Pierce, Rockford, IL) and a chromogenic histochemical reaction (NovaRed, Vector Laboratories, Burlingame, CA). Sections were counter-stained with Mayer’s hematoxylin (Sigma, St. Louis, MO).

### Cell Culture

HEK cells were maintained in DMEM supplemented with 10% FBS and 50 µg/mL of each penicillin and streptomycin and maintained at 37°C in a humidified, 5% CO_2_ incubator. MN9D cells [48] (a gift from Dr. Alfred Heller, University of Chicago) were maintained in DMEM high glucose, supplemented with 10% FBS and 50 µg/mL of each penicillin and streptomycin and maintained at 37°C in a humidified, 5% CO_2_ incubator. Cells were grown to 80% confluence and transfected with 2 µg plasmid using Lipofectamine 2000 (Invitrogen, Carlsbad, CA). The following day, cells were transferred to Poly-D coated glass coverslips and allowed to adhere overnight. Cells were fixed in 4% PFA, washed in 1X PBS, counterstained with DAPI and mounted to slides for imaging.

### Morpholino Knockdown of *tor1*

To examine the function of *tor1* during development we employed morpholino oligonucelotides (MOs) to target *tor1* expression. MOs bind complementary nucleic acid sequences with high affinity, allowing sequence-specific inhibition of splicing or translation by targeting splice consensus sequences in pre-mRNA or the translational initiation sequences of mRNAs [49]. 6 ng each of two different *tor1* splice-blocking MOs (*exon2/intron2*: 5′-ATATGAAGTCAGCTTACCTTGTAGG-3′; *exon3/intron3*: 5′-TGTTAGTAGACACTGACCTGAGGAA-3′) or two different controls (*standard*: 5′-CCTCTTACCTCAGTTACAATTTATA-3′; *random:* 5′-(N)_25_-3′) were diluted in 2 nL 1× Danieau buffer (58 mM NaCl, 0.7 mM KCl, 0.4 mM MgSO_4_, 0.6 mM Ca(NO_3_ )_2_, 5.0 mM HEPES, pH 7.6) and injected into single cell embryos. Embryos were mechanically dechorionated at 24 hours post-fertilization (hpf) and raised at 28.5°C on a 14∶10 light:dark cycle for subsequent behavioral experiments.

### Spontaneous Movement Assays

Spontaneous movement was measured at 5 dpf as previously described [50], [51]. Embryos (at least 16 per group) were transferred to individual wells of a 96-well plate. Spontaneous movement was recorded using a video camera, and the resulting video files were analyzed using custom Matlab® algorithms [50] to determine: mean velocity (total displacement/total time of recording); active velocity (total displacement/time spent moving); % time moving (time spent moving/total time of recording); mean rest duration and mean duration of active episodes. All data sets were analyzed by both parametric (ANOVA) and non-parametric (Kruskal-Wallis) tests. Most of the data sets at this age point were normally distributed as evaluated by D’Agostino & Pearson’s test. For clarity, raw data are shown along with mean and standard error.

## Results

### Identification of a Zebrafish TorsinA Homologue

**Figure 1:**
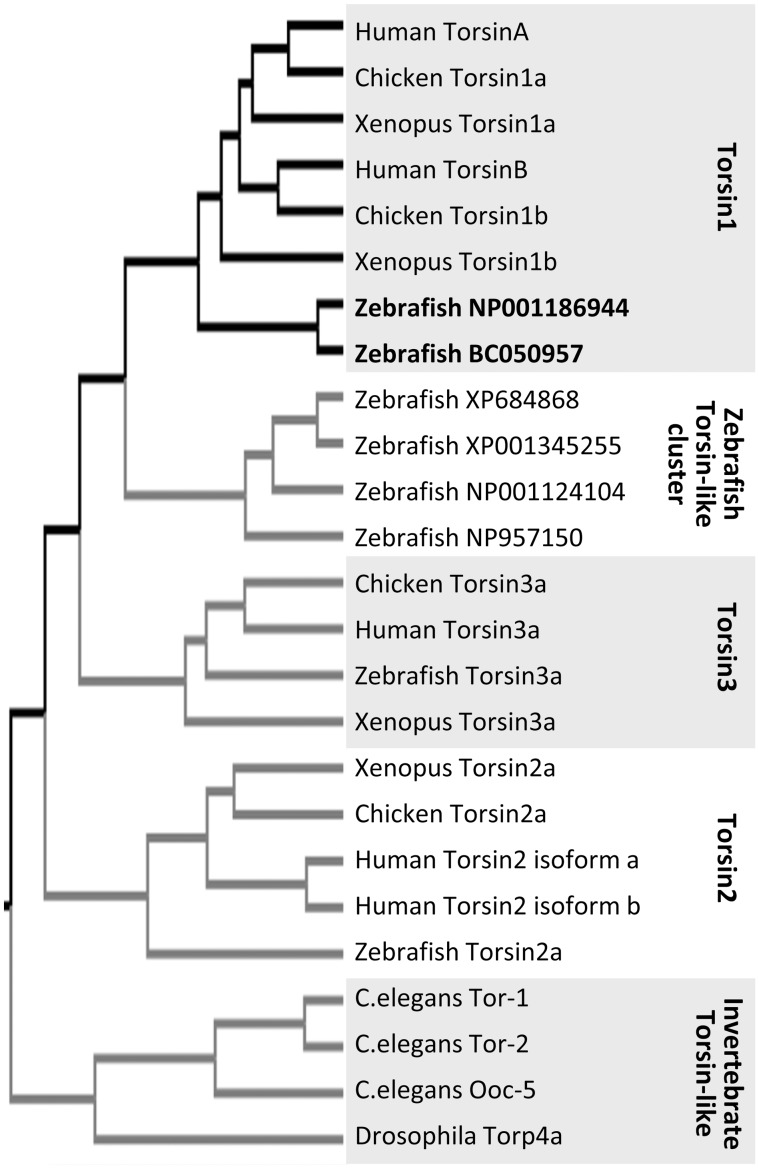
Identification of zebrafish Torsins. Human TorsinA was used as a probe to interrogate the NCBI zebrafish RefSeq and non RefSeq RNA databases by TBLASTN. Eight different zebrafish mRNA and predicted mRNA sequences with homology to TorsinA were translated and aligned with Torsins from *C. elegans*, *D. melanogaster*, Xenopus, Chicken, and Human. A dendrogram was constructed to illustrate the inferred evolutionary relationships between Torsin family proteins from different species.

**Figure 2:**
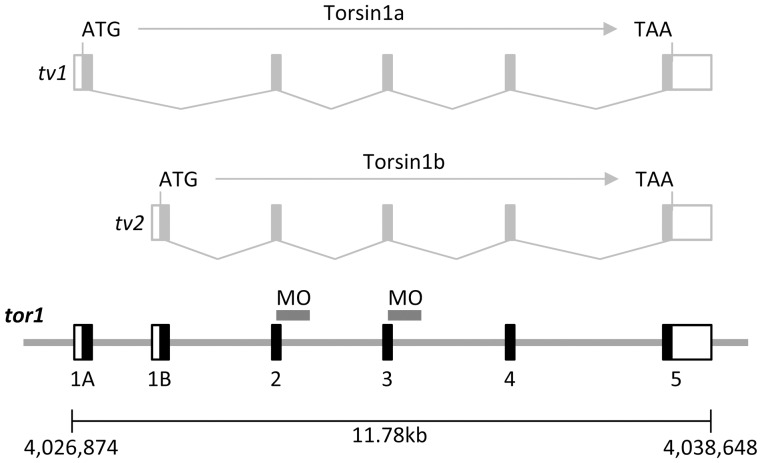
Zebrafish *tor1* is expressed as two different transcript variants. The two zebrafish transcripts most closely related to human TOR1A were found to be transcribed from a single gene by inclusion of alternative 5′ exons. *tor1* transcript variants 1 and 2 encode Torsin1 isoforms a and b, which differ at their N termini. The two transcripts are depicted above the genomic sequence. The splice boundaries targeted by morpholino oligonucleotides in figure 10 are labeled ‘MO’.

To identify zebrafish proteins with homology to human TorsinA, the zebrafish RefSeq and non RefSeq mRNA databases (www.ncbi.nlm.nih.gov; final accession date: August, 11, 2011) were interrogated by TBLASTN using the human TorsinA protein sequence as a probe. Eight sequences (six transcripts and two predicted transcripts) with homology to human TorsinA were further analyzed. The predicted translations of the zebrafish sequences were aligned with Torsin protein sequences derived from human, chicken and xenopus, as well as the nearest *C. elegans* and drosophila homologs, using the ClustalX 2.1 algorithm, [52], and a dendrogram was constructed to illustrate the inferred evolutionary relationships between the proteins, using the BEAST program [53] (figure 1). Two putative zebrafish proteins appeared most closely related to Torsin1 family proteins from other vertebrate species. Putative zebrafish orthologues of mammalian Torsin2 and Torsin3 family proteins were also detected. Comparison of the mRNA and genomic sequences for the two putative zebrafish Torsin1 proteins showed that the two transcripts are derived from a single gene by inclusion of alternative 5′ exons (figure 2). We refer to the gene (si:ch73-178d14.1) as *tor1*, and its two transcript variants as *tor1_tv1* (NM_001200015) and *tor1_tv2* (BC_050957). The resulting proteins are referred to as Torsin1a and Torsin1b respectively. The *tor1* gene maps to zebrafish chromosome 21, and spans approximately 11.78 kb of sequence. An expressed sequence tag (EST; AA542632) encoding amino acids 14–140 of Torsin1a was previously reported as *TOR1C* [54].

The zebrafish genome also contains an additional cluster of four adjacent genes on chromosome 23 predicted to encode Torsin-like proteins. Sequence comparison suggests that these putative proteins are most closely related to Torsin1 and Torsin3 family proteins, although they show less homology than either Torsin1a or Torsin1b to human TorsinA. We found evidence that two of these genes (NP_001124104 and NP_957150) are transcribed. Each of these genes shows divergent exon structure with respect to human TOR1A, suggesting that they are more distantly related than *tor1* to TOR1A (see below). The Torsin-like gene cluster was not found in other teleosts and was located in a region of chromosome 23 with little conservation of synteny with respect to other teleosts (not shown).

**Figure 3:**
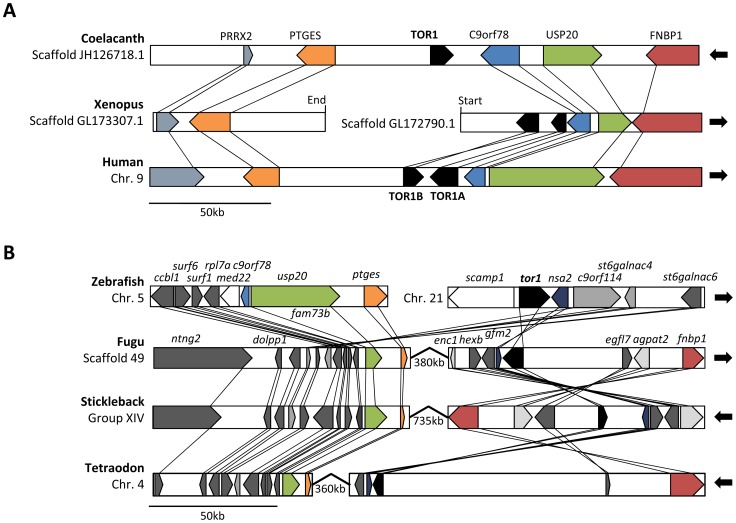
**A:** Syntenic relationships of *tor1*, TOR1A and TOR1B. The diagram shows the order and orientation of genes flanking TOR1A and TOR1B in the human and xenopus genomes, and *tor1* in the coelacanth genome. The map is shown to scale; large arrows show the direction of the telomere. TOR1A, TOR1B and *tor1* are colored black. The flanking genes are colored green (USP20), red (FNBP1), blue (C9orf78), orange (PTGES) and lilac (PRRX2). Orthologous genes are connected by solid lines. **B:** The diagram shows the order and orientation of genes flanking *tor1* in the zebrafish, fugu, stickleback, and tetraodon genomes. The color scheme is identical to panel A; additional flanking genes are colored as follows: dark grey - conserved chromosomal positions in all four fish species; light grey - conserved chromosomal positions in two or three fish species; white - chromosomal position not conserved.

In order to better understand the relationships between zebrafish and human Torsins, we next examined the genomic context of Torsin1 family genes in the human, and how this is conserved in other vertebrate species (figure 3). In human, the dual torsin1 family members TOR1A and TOR1B are adjacent and inverted with respect to one another. The order of the flanking genes PRRX2– PTGES – TOR1A – TOR1B – C9orf78– USP20– FNBP1 is conserved from mammals to amphibians (figure 3A). In coelacanth, a later diverging fish species, this gene order is also conserved. However, similar to other fish species, coelacanth has a single *tor1* gene instead of the dual TOR1A and TOR1B genes found in xenopus and human. This suggests that *tor1* was duplicated at the root of the tetrapod lineage.

In earlier diverging fish species (figure 3B), substantial genomic rearrangements with respect to tetrapods and coelacanth disrupt the conservation of synteny surrounding the Torsin1 family genes. *fnbp1* is found in close proximity to *tor1* in fugu, stickleback and tetraodon, but is located ≈2 MB upstream of *tor1* in the zebrafish genome. Interestingly, the genes immediately flanking TOR1A and TOR1B in human are found in syntenic blocks separated from *tor1* in earlier-diverging fish species. Thus *ptges* and *usp20* are adjacent to one another, but located >350 kb from *tor1*, in fugu, stickleback and teraodon; in zebrafish, these genes and *c9orf78* are located on a different chromosome to *tor1*. However, *tor1* otherwise shows conserved syntenic relationships in all four fish species. Given the proximity of *tor1* and *fnbp1* in three of the species, and the single *tor1* gene in coelacanth despite conservation of synteny with human a TOR1A and TOR1B, these data are consistent with the other findings reported here suggesting that zebrafish *tor1* and human TOR1A and TOR1B may share a common genomic origin.

### *tor1* Transcriptional Start Sites, Full cDNA Sequences, Transcript and Genomic Organization

**Figure 4:**
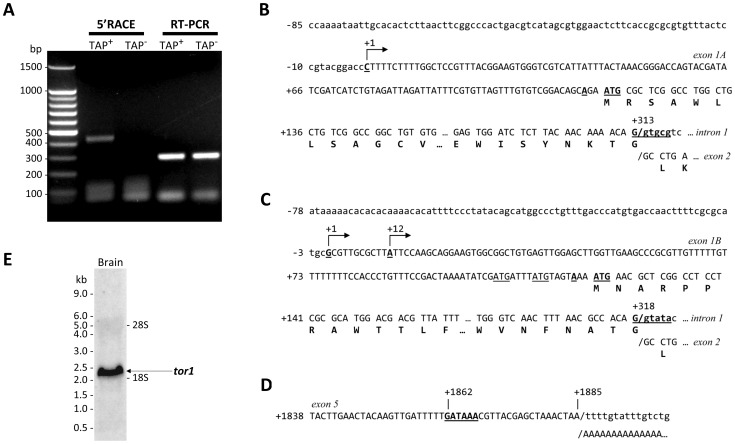
*tor1* promoters and transcripts. **A:** Zebrafish brain total RNA was treated with tobacco acid pyrophosphatase (TAP^+^; lanes 2 and 4) or untreated (TAP^−^; lanes 3 and 5) prior to RACE adapter ligation and reverse transcription. *tor1* was amplified by 5′RACE using a *tor1* exon 2 reverse primer and a RACE adapter primer (lanes 2 and 3) or by RT-PCR using *tor1* primers (lanes 4 and 5). **B, C:** The transcriptional start sites of *tor1_tv1* (B) and *tor1_tv2* (C) were determined by comparing the genomic sequence with sequences of the 5′ RACE products shown in panel A. The transcriptional start sites are shown underlined with arrows; bases are numbered such that +1 represents the first nucleotide of the most 5′ transcriptional start site. The open reading frames of exons 1A and 1B and their 3′ splice sites are shown. The consensus **A**NN**AUG** translational initiation signals are indicated in bold and underlined; non-consensus AUG sequences in the 5′UTR of *tor1_tv2* are underlined. **D:** 3′RACE was employed to determine the 3′ terminus of the transcript. The polyadenylation signal within exon 5 is underlined in bold. The position of the poly(A) tail in the mRNA is indicated. **E:** Brain total RNA was separated electrophoretically and the resulting northern blot was probed using a cRNA probe to *tor1*. The positions of molecular size standards and the 28S and 18S rRNA bands are shown.

We cloned zebrafish *tor1* cDNA and mapped its transcriptional start sites by rapid amplification of cDNA ends (RACE), using RNA isolated from adult zebrafish brain tissue. 5′RACE was carried out using the tobacco acid pyrophosphatase (TAP) method to allow specific ligation of the RACE adapter to the 5′ end of mRNA transcripts (figure 4A). *tor1* cDNA was amplified using a 3′ primer complementary to exon 2 of the *tor1* mRNA (which is shared by both transcript variants) and a 5′ RACE adaptor primer, yielding a single PCR product. TAP^−^ controls confirmed that amplification of this product was dependent on hydrolysis of the 7-methylguanylate mRNA cap prior to adapter ligation; RT-PCR controls confirmed that reverse transcription was equivalent in both TAP^−^ and TAP^+^ samples. These controls show that the boundary between the RACE adapter and the cDNA sequence corresponds to the 5′ end of the transcript and hence the transcriptional start site in the genomic sequence. 5′RACE products were cloned and sequenced: *tor1_tv1* and *tor1_tv2* were found to initiate transcription from different promoters located at the 5′ end of the gene; the unique first exons in each transcript splice into a common mRNA at exon 2 (figure 2). A single major transcriptional start site was found in *tor1_tv1*, whereas *tor1_tv2* showed two different 5′ termini (figure 4B, C). As is commonly found in ubiquitously-expressed genes, neither promoter region contained a consensus TATA box or initiator motif [55].

The 3′ end of the *tor1* transcript was cloned by 3′RACE (figure 4D). A single polyadenylation signal (GATAAA) was located at position 1862 with respect to the first nucleotide of *tor1_tv1*. This poly(A) signal is located 24 bp upstream of the poly(A) tail, and is shared by both transcript variants. After allowing for addition of the poly(A) tail, the sizes of the complete *tor1_tv1* (1881 bp) and *tor1_tv2* (1886 bp) cDNA sequences are compatible with the single ≈2.1 kb *tor1*-hybridizing band found on northern blot analysis of brain RNA (figure 4E).

**Figure 5:**
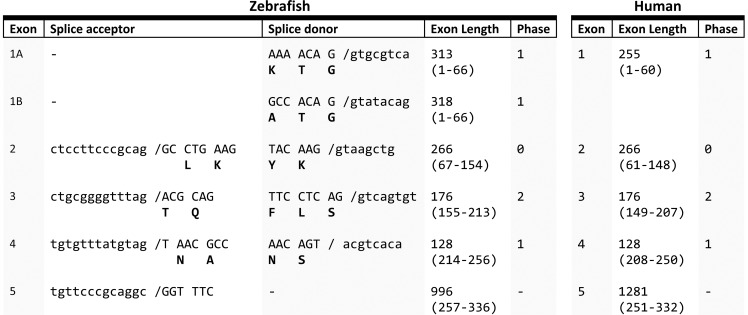
Genomic organization of zebrafish *tor1* compared with human TOR1A. The splice acceptor and donor sequences for each intron/exon boundary of the zebrafish *tor1* gene are shown, alongside the length of each exon and the phase of each splice boundary. The exon length and phase of human TOR1A are shown for comparison.

The *tor1* cDNA sequences were next mapped to the Zv9 assembly of the zebrafish genomic sequence and the exon boundaries and splice sites determined (figure 5). Both zebrafish *tor1* and human TOR1A transcripts contain 5 exons. The phases of all splice boundaries are conserved between TOR1A and *tor1.* In addition, exons 2, 3, and 4, which encode the majority of the open reading frame, share identical lengths between the human and zebrafish sequences. This striking conservation of genomic organization between the human TOR1A and zebrafish *tor1* genes provides further evidence that they descend from a common ancestral gene.

### Expression of *tor1* in Zebrafish

**Figure 6:**
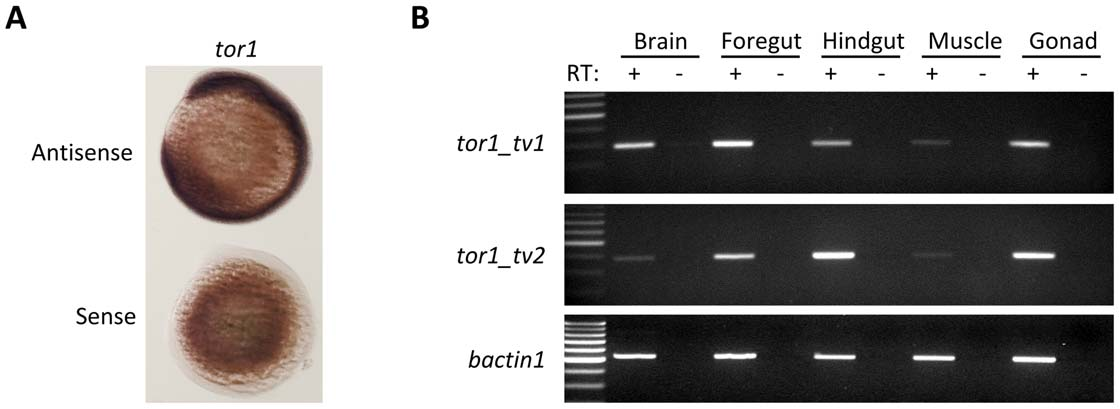
*tor1* is expressed ubiquitously. A: RNA i*n situ* hybridization was employed to detect the *tor1* transcript during development. Hybridized probe was detected using a histochemical reaction with a blue/purple product. The photomicrograph shows embryos at 12 hpf; the upper of the pair was hybridized with a *tor1* antisense probe and the lower with a *tor1* sense control probe. **B:** Reverse transcriptase PCR was employed to detect *tor1_tv1* (upper panel) or *tor1_tv2* (middle panel) mRNA in brain, foregut, hindgut, muscle, and gonads of adult zebrafish. Total RNA was treated with reverse transcriptase (RT^+^); controls lacking reverse transcriptase (RT^-^) excluded amplification of genomic DNA sequences. *bactin1* was amplified as a ubiquitously expressed control mRNA (lower panel).

We next determined the temporal and spatial expression patterns of *tor1*. mRNA for both *tv1* and *tv2* was detected by RT-PCR during early embryonic development (not shown). However, isoform-specific probes complementary to the short exon sequences unique to each transcript yielded insufficient signal in whole mount RNA *in situ* hybridization assays to localize *tor1* transcripts. Consequently, we employed a larger cRNA probe complementary to the entire *tor1* open reading frame (ORF) shared by both transcripts. This *tor1* ORF cRNA probe specifically detects a single *tor1* transcript on Northern blot (figure 4E) and shares less than 60% sequence identity with the closest related gene; consequently, the probe is expected to hybridize specifically with *tor1* transcripts under the high stringency conditions used for RNA *in situ* hybridization. At 12 hours post fertilization (hpf), *tor1* was expressed throughout the embryo (figure 6A). A sense control probe did not show hybridization at this time point, suggesting that the observed staining pattern was not attributable to non-specific hybridization. At later developmental points, the pattern appeared unaltered, but the expression level progressively reduced and was barely detectable by 96 hpf. In the adult, both *tor1_tv1* and *tor1_tv2* were detected by RT-PCR in several tissues including brain, foregut, hindgut, muscle, and gonad (figure 6B). Relative to *bactin1*, expression of both isoforms was lower in brain and muscle compared to gut and gonad, suggesting reduced expression of *tor1* in post-mitotic tissues. In addition, the relative expression of the two transcripts differed between tissues. For example, *tor1_tv1* was more abundant in foregut, whereas *tor1_tv2* was expressed more strongly in the hindgut. These data suggest that expression of the two transcripts is regulated independently by distinct *cis*-regulatory elements, as might be predicted from their separate promoters.

### Zebrafish Torsin1 Proteins and their Subcellular Localization

**Figure 7:**
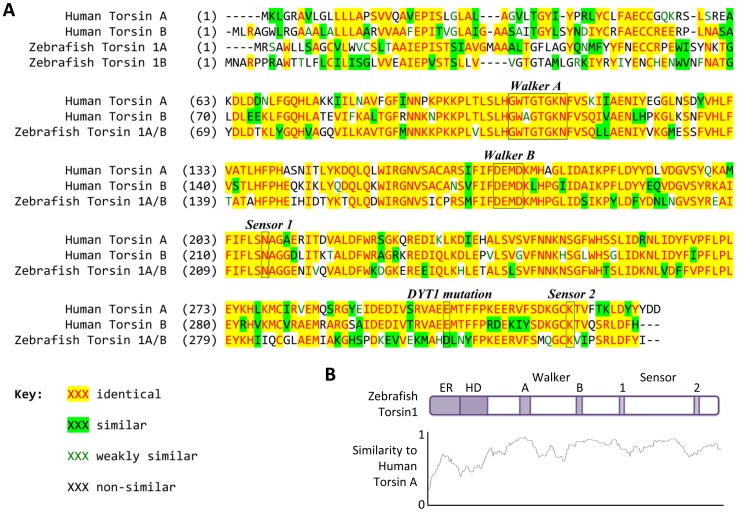
Zebrafish and human Torsin1 family proteins are highly homologous. **A:** The AlignX implementation of the ClustalW algorithm was used to align the amino acid sequences of zebrafish Torsin1a and 1b, and human TorsinA and TorsinB. The Walker A (ATP binding), Walker B (ATP hydrolysis), Sensor 1 and Sensor 2 domains are labeled. The position of the glutamic acid deletion associated with DYT1 dystonia is indicated. Identical and similar amino acids at each position of the alignment are colored as shown. **B:** Zebrafish Torsin1a is depicted schematically showing the functional domains discussed in the text. The graph below shows similarity between zebrafish Torsin1a and human TorsinA at each position of a sliding 15-amino acid window of comparison, corresponding to the schematic drawing of Torsin1 above (0 = no homology, 1 = identity). Key: ER, endoplasmic reticulum signal; HD, hydrophobic domain.

The amino termini of Torsin1a and Torsin1b, encoded by exons 1A and 1B, are divergent. However, both are predicted to contain a cleavable ER retention signal, followed by an alpha-helical domain and both Torsin1a and Torsin1b are 336 amino acids in length. Alignment of zebrafish Torsin1a and Torsin1b with human TorsinA and TorsinB revealed conservation of key functional domains, including consensus sequences for the ATP-binding Walker A domain, the ATP-hydrolysis Walker B domain, and the Sensor 1, and Sensor 2 domains (figure 7A). Both zebrafish isoforms are 59% identical and 78% similar to TorsinA and 57% identical and 77% similar to TorsinB. Human TorsinA and TorsinB share 66% identity and 77% similarity. The highest degree of divergence between zebrafish Torsin1 and human TorsinA was observed within the amino terminus (figure 7B). Similarly, maximal divergence between Human TorsinA and TorsinB was also within the amino terminus.

**Figure 8:**
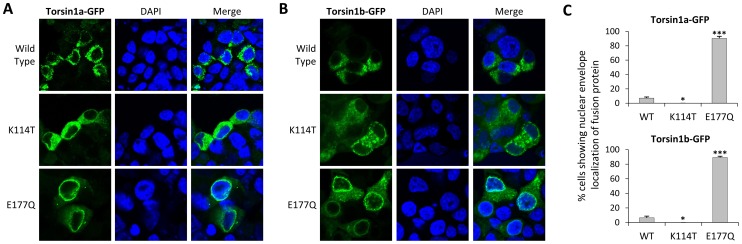
ATP-binding and ATP-hydrolysis mutations in Torsin1 alter cellular localization. MN9D cells were transfected with expression plasmids encoding either (A) Torsin1a-eGFP or (B) Torsin1b-eGFP fusion proteins. The images show single confocal planes through transfected cells demonstrating GFP fusion protein localization (green, left column) relative to the nucleus (DAPI counterstain, blue, center column). For each panel, the top row of images shows wild type Torsin1, the middle row of images shows a Torsin1[K114T] mutant, which disrupts the Walker A (ATP-binding) domain, and the bottom row shows a Torsin1[E177Q] mutant, which disrupts the Walker B (ATP hydrolysis) domain. (C) Four independent samples for each construct were evaluated for nuclear envelope localization of the fusion protein. The graphs show the mean % cells with nuclear envelope localization (100–200 cells were scored for each construct; error bars show standard error; ***P<0.0001, *p<0.05 versus WT, heteroscedastic 2-tailed T-test with Bonferroni correction).

The DYT1 dystonia mutation causes deletion of one glutamate residue from an EE motif in the carboxyl terminal of TorsinA. Although the pathophysiological properties of this mutation are yet to be fully elucidated, one striking property of the mutant is relocalization of the protein from the endoplasmic reticulum to the nuclear envelope [12]. The homologous region in zebrafish Torsin1 contains the amino acids HD (figure 7A), precluding mutagenesis studies to determine whether the zebrafish protein undergoes similar relocalization following introduction of the same mutation. However, redistribution of human TorsinA to the nuclear envelope is also observed when null mutations are introduced into the Walker B ATP hydrolysis domain of TorsinA [9], [12]. To investigate whether the subcellular localization of Torsin1a and Torsin1b can be altered by similar mutations, we constructed zebrafish Torsin1-eGFP fusion proteins and examined their localization in transfected cells. Both Torsin1a and Torsin1b isoforms primarily localized to the cytosol (figure 8A, B) in the mouse dopaminergic MN9D cell line. In a small percentage of cells, Torsin1-eGFP fusion protein could also be detected at the nuclear envelope (figure 8C). This distribution pattern is consistent with the diffuse intracellular localization observed with human TorsinA [11]. Next, we examined whether mutations in the conserved ATP binding (Walker A) and ATP hydrolysis domains (Walker B) of Torsin1 altered the cellular redistribution of Torsin1. Similar to human TorsinA, mutation (K114T) of the ATP binding domain of Torsin1 [13], [56] did not alter the cytoplasmic localization of the Torsin1-eGFP fusion proteins (figure 8A, C). However, Torsin1-eGFP[K114T] was not detected in the nuclear envelope of transfected cells, suggesting that ATP binding is required for Torsin1 to enter the nuclear envelope. Conversely, mutation (E177Q) of the ATP hydrolysis domain [13], [56] caused accumulation of Torsin1-GFP in the nuclear envelope (figure 8B, C). These results show that (i) similar to human TorsinA, zebrafish Torsin1 can enter the nuclear envelope; and (ii) mutation of the ATP hydrolysis domain of Torsin1 results in its sequestration in this location. Zebrafish Torsin1 proteins thus share key aspects of dynamic cellular localization with human TorsinA, and this depends on at least some of the same functional domains. This suggests that zebrafish Torsin1 and human TorsinA may share a common set of interacting proteins.

### Zebrafish Torsin1 Proteins – glycosylation and Expression in Adult Brain

**Figure 9:**
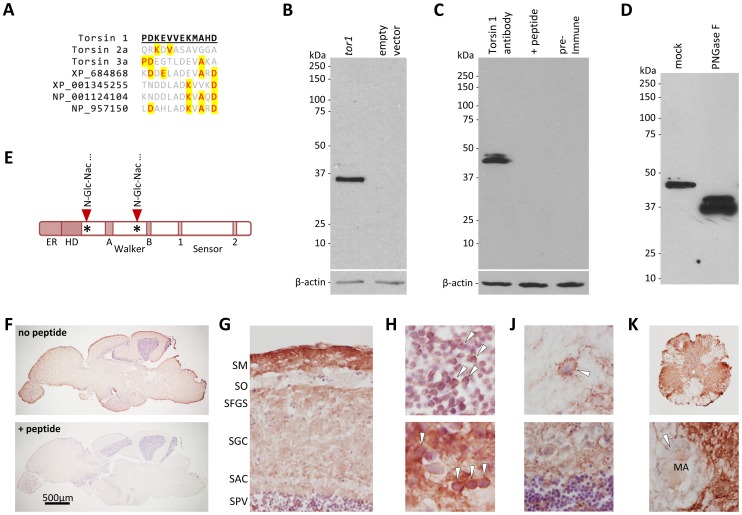
Torsin1 is a neuronal glycoprotein. **A:** The peptide sequence used to raise polyclonal antibodies to zebrafish Torsin1 is shown underlined in bold. An alignment between Torsin1 and the homologous regions of other zebrafish Torsin-like proteins is shown to illustrate the lack of potentially cross-reacting sequences in other Torsins. Amino acids that are identical to Torsin1 at each position are highlighted in yellow. **B:** HEK cells were transfected with a plasmid encoding Torsin1b (lane 1) or with empty vector (lane 2). A western blot made with lysates from transfected cells was probed with affinity-purified Torsin1 antibody. The blot was then re-probed with an antibody to β-actin as a control for equal protein loading (lower panel). **C:** Identical samples of zebrafish whole brain lysate were resolved by SDS-PAGE in adjacent lanes of a gel and the resulting western blot was divided into strips each containing a single lane. The strips were probed with either affinity-purified Torsin1 antibody (lane 1), affinity-purified Torsin1 antibody pre-incubated with the peptide immunogen shown in panel A (lane 2), or pre-immune serum (lane 3). The blots were then re-probed with an antibody to β-actin as a control for equal protein loading (lower panel). **D:** Whole brain lysate was either treated with PNGase F (lane 2) to remove N-linked carbohydrate moieties, or mock treated without addition of PNGase F (lane 1), prior to SDS-PAGE and immunoblotting with Torsin1 antibody. **E:** Schematic representation of Torsin1 showing the positions of the consensus N-glycosylation sites. **F–K:** Adult zebrafish CNS sections were labeled with Torsin1 antibody; immunoreactive structures were revealed by chromogenic histochemistry with a red reaction product. Sections were counterstained with Mayer’s hematoxylin so that nuclei appear blue. (F) Parasagittal sections of the whole zebrafish brain are shown labeled with Torsin1 antibody. In the lower panel, the antibody was pre-incubated with the peptide immunogen. (G) Optic tectum. Key: SM, stratum marginale; SO, stratum opticum; SFGS, stratum fibrosum griseum superficiale; SGC, stratum griseum centrale; SAC, stratum album central; SPV, stratum periventriculare. (H) Periventricular layer of optic tectum (upper panel) and medulla oblongata (lower image), showing cytoplasmic Torsin1 expression in neurons (arrowheads). (J) Thalamus (upper panel) and cerebellum (lower panel) showing Torsin1-related immunoreactivity in neuropil; arrowhead in upper panel shows a neuron surrounded by punctate Torsin1 immunoreactivity that may be present in nerve terminals. (K) Spinal cord at low magnification (upper panel) and high magnification (lower panel) showing robust Torsin1 immunoreactive signal in gray matter, but absence of Torsin1 in white matter. The arrowhead in the lower panel shows the myelin sheath surrounding the Mauthner axon (MA).

In order to better characterize Torsin1, we next developed a polyclonal antibody against a peptide sequence near the carboxyl terminal, which is divergent between Torsin1 and other zebrafish Torsins (figure 9A). Polyclonal antisera to this antigen were raised in rabbits, and affinity-purified against the peptide. The purified antibody was initially tested by probing western blots of protein lysates from HEK293 cells, which were transfected with a construct expressing *tor1_tv2* under the CMV immediate-early promoter, or empty vector as a negative control (figure 9B). A single immunoreactive species of 35 kDa was detected in cells expressing *tor1_tv2*, whereas no immunoreactive proteins were present in cells transfected with empty vector. This demonstrates that the antibody can recognize Torsin1 expressed in cultured cells. Next, we analyzed adult zebrafish brain lysate by western blot (figure 9C). Two bands were detected with apparent molecular weights of 47 kDa and 45kDa. Detection of both bands was prevented by pre-incubation of the antibody with the cognate peptide, and neither band was present when blots were probed with pre-immune serum. These data confirm that the 47 kDa and 45 kDa species contain the cognate peptide sequence, and therefore most likely represent Torsin1. *tor1* is expressed at low levels in embryos, and we did not detect convincing Torsin1-immunoreactive signal during early development. Consequently we were not able to further confirm specificity of the antibody for Torsin1 *in vivo* by showing loss of immunoreactive signal after morpholino knockdown of *tor1* expression (see discussion). The apparent molecular weight of Torsin1 differed between cultured cells and the zebrafish brain *in vivo*. It is known that Torsins are subject to post-translational modifications, including signal peptide cleavage and N-glycosylation [11], [57]. Pre-incubation of brain lysate with peptide:N-glycosidase F (PNGase F), an amidase that cleaves between asparagine and the innermost N-acetyl-glucosamine (GlcNAc) residue of the oligosaccharide chain of N-linked glycoproteins, altered the electrophoretic mobility of the Torsin1-immunoreactive species, which migrated at 36–38 kDa following PNGaseF treatment (figure 9D). This mobility shift demonstrates the presence of N-linked carbohydrate chains totaling approximately 9 kDa. The consensus ‘sequon’ or target sequence for N-glycosylation is N-X-S or N-X-T, where X is any residue except for proline. There are two predicted N-glycosylation sites in Torsin1, at residues 63 and 164 (figure 9E). The lower apparent molecular weight observed in cultured cells suggests that post-translational processing of Torsin1 differs between transformed human kidney cells and the zebrafish brain *in vivo*.

Sections from adult zebrafish CNS were labeled with Torsin1 antibody. Immunoreactive signal was seen in multiple different brain regions and was abolished by pre-incubation of the antibody with the cognate peptide (figure 9F), indicating that the tissue epitope recognized by the antibody is shared by the peptide, and is therefore likely to represent Torsin1. Torsin1-related immunoreactivity was abundant in grey matter regions, such as the optic tectum, cerebellum, brainstem nuclei and central grey matter of the spinal cord (figure 9F–K). In contrast, white matter tracts, including the optic nerves and tracts, the medial longitudinal fasciculus and long descending tracts of the spinal cord, did not show Torsin1-related immunoreactivity (figure 9G, K). Cytoplasmic labeling was apparent in many neurons of the brainstem, optic tectum and diencephalon (figure 9H). Torsin1-related immunoreactivity was also abundant in the neuropil and some neurons showed punctate staining around the perimeter of their cytoplasm, raising the possibility that Torsin1 may also be expressed in nerve terminals (figure 9J). These data suggest that Torsin1 is expressed in the cell body, dendrites and possibly also terminals of neurons in multiple CNS regions, but is not expressed in myelinated fibers.

### Physiological Expression of tor1 is not Required for Early Development of Motor Circuits

**Figure 10:**
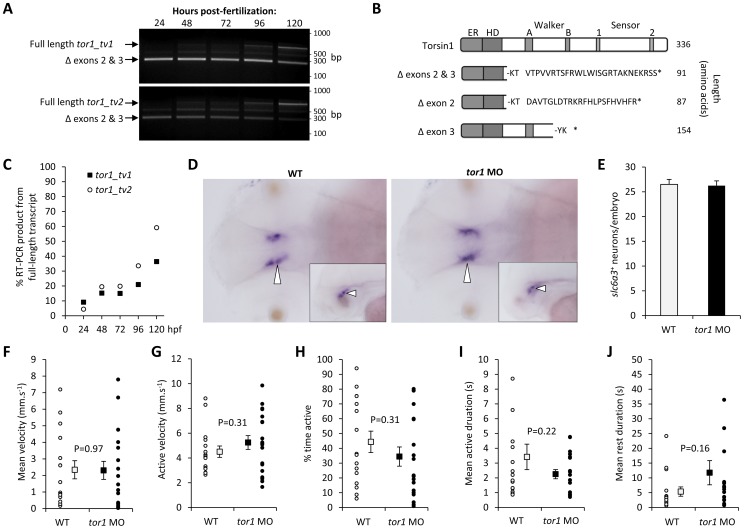
Torsin1 is non-essential for early development of the motor system. **A:** Single-cell zebrafish embryos were microinjected with morpholino oligonucleotides designed to block splicing of *tor1* exons 2 and 3 (see figure 2). At the times indicated between 24 and 120 hours post-fertilization, RNA was harvested from developing zebrafish and subjected to RT-PCR using primers specific for *tor1_tv1* (upper panel) or *tor1_tv2* (lower panel). The pictures show ethidium bromide stained agarose gels after the PCR products were resolved by electrophoresis. The positions of PCR products corresponding to wild-type *tor1* mRNA and the transcript lacking exons 2 and 3 are shown. **B:** A schematic representation is shown of the predicted protein products produced by transcripts lacking exons 2, 3 or both exons. It is not known if any of these truncated products is stable. **C:** The % *tor1* RT-PCR product arising from the wild-type, full-length mRNA at each time point following MO injection was estimated by densitometry. **D:** Whole mount RNA *in situ* hybridization was carried out using a cRNA probe for *slc6a3* (‘*dat*’) to localize dopaminergic neurons. Representative dorsal views are shown of the brains of an uninjected control embryo (left panel) and an embryo lacking Torsin1 (right panel) at 48 hours post-fertilization. The inset panels show representative lateral views. Developing dopaminergic neurons in the diencephalon are indicated by arrowheads. **E:** The total number of *slc6a3* ^+^ cells in the diencephalon was counted in zebrafish lacking Torsin1 and control zebrafish at 48 hpf. Graphs show mean number of cells, error bars show standard error. **F–J:** Spontaneous movements of uninjected wild-type zebrafish (n = 16) and zebrafish injected with MO targeting *tor1* (n = 19) were recorded at 5 dpf in 96-well plates using a video camera. Recordings were analyzed to determine: (F) mean velocity (total displacement/total time of recording); (G) active velocity (displacement/time spent moving); (H) % time active; (I) mean duration of active episodes; and (J) mean duration of rest episodes. For each graph, values for each individual larva are shown as small circles and the mean for the group shown as a filled square. Error bars shown standard error.

To determine the function of *tor1* during larval development, we targeted its expression by using morpholino antisense oligonucleotides (MOs). We opted to use splice site MOs to inhibit pre-mRNA processing, because knockdown could be verified readily by RT-PCR. Initial studies showed that a combination of two MOs targeting the exon2/intron2 and exon3/intron3 splice donor consensus sequences caused both exons 2 and 3 to be excluded from the transcript (figure 10A). This resulted in a frame shift and formation of a premature stop codon, which abrogated expression of the Walker B domain and both Sensor domains (figure 10B). This combination of MOs efficiently disrupted expression of *tor1* mRNA up to up to 4 days post fertilization (dpf); densitometry estimated that less than 10% of the *tor1* RT-PCR product at 24 hpf was derived from the full-length transcript (Figure 10C). To exclude phenotypes attributable to adverse effects from microinjection, and non-specific toxicity of MOs, we compared *tor1* knockdown zebrafish with wild-type animals, and with animals injected with control non-targeting MO.

There were no differences between uninjected, control MO injected, and *tor1* MO injected embryos in any of the parameters we measured. For clarity, data are shown for WT and *tor1* knockdown animals only. Zebrafish from all three experimental groups showed normal morphological development through day 7 (not shown). We next evaluated development of dopaminergic neurons, because the function of the dopamine system is disrupted in some patients with dystonia [17]. Whole mount RNA *in situ* hybridization was employed to label developing dopaminergic neurons using a probe specific to the *slc6a3* transcript encoding the dopamine transporter, which is expressed in dopamine neurons [58], [59]. We did not observe any differences between wild type larvae and *tor1* morphant larvae in the position of dopaminergic neurons or in the intensity of *slc6a3* expression at 48 hpf or 72 hpf (figure 10D and data not shown). The total number of diencephalic *slc6a3^+^* neurons was determined in each population of zebrafish; knockdown of *tor1* did not affect the number of dopamine neurons at 48 or 72 hpf (figure 10E and data not shown). We conclude that, similar to murine TOR1A^−/−^ lines [22], knockdown of *tor1* in zebrafish did not give rise to abnormalities in the number or position of dopamine neurons during development.

Finally, we asked whether knockdown of *tor1* provoked abnormalities of motor function by employing a previously described assay [50], [51] to quantify spontaneous propulsive movements in uninjected larvae, control MO injected larvae, and Torsin1 morphant larvae. Compared with controls, larvae lacking physiological expression levels of Torsin1 did not show statistically significant differences in mean velocity, percent time moving, active velocity, mean duration of movement bursts, or mean rest duration between movements at 4–6 dpf (figures 10F-J show data at 5 dpf). At earlier developmental time points before 4 dpf, when *tor1* knockdown was most prominent, larval propulsive movements are infrequent and variable, making it difficult to detect differences between experimental groups [51]. To evaluate zebrafish between 1–4 dpf, we employed a second method that determines changes in pixel grayscale values from frame to frame of a video stream, rather than detecting displacement of the larval centroid. The pixel quantification method is sensitive to both propulsive and non-propulsive movements, such as coiling and re-orientation movements, and consequently has previously allowed us to detect differences in motor activity between experimental groups at early developmental points [60]. However, even using this method, we did not detect differences between the motor activity of controls and *tor1* knockdown animals between 1–4 dpf (data not shown). Taken together, these findings suggest that wild-type expression levels of Torsin1 are not required for early morphological development and function of the motor system.

## Discussion

The data presented here suggest that a novel zebrafish gene, *tor1,* is a single zebrafish homolog of the mammalian TOR1A and TOR1B genes. Conservation of sequence and genomic organization provide strong evidence that the zebrafish and human genes share a common ancestor, and we found notable similarities between the expression patterns of the human and zebrafish mRNAs, and the domain organization and subcellular localization of the proteins. There is limited conservation of synteny between teleost and tetrapod genomes in the immediate vicinity of the *tor1* genes, suggesting that substantial genomic reorganizations occurred in this region during evolution. One of the more striking differences is the presence of adjacent TOR1A and TOR1B genes in tetrapods, whereas the genomes of zebrafish, fugu, stickleback, tetraodon and coelacanth all contain a single *tor1* gene. These data strongly suggest that an ancestral *tor1* locus was duplicated during the fin-to-limb transition, resulting in the occurrence of distinct TOR1A and TOR1B genes before the emergence of amphibious tetrapods. Whether the separate TOR1A and TOR1B genes have divergent functions remains to be determined; TorsinA and TorsinB show complementary expression patterns [61] and it is possible that they mediate similar cellular functions in different groups of cells, similar to the numerous other paralogous proteins with distinct expression patterns that show redundant functions [62]. In support of the possibility that TorsinA and TorsinB have redundant functions, there is evidence that TorsinB may partially compensate for the functional effects of mutations in TorsinA: morphological abnormalities of the nuclear envelope in fibroblasts derived from ΔE/ΔE knock-in mice were exacerbated by loss of TorsinB [61]. Consequently, it is possible that the single *tor1* gene in zebrafish will prove advantageous for determining the role of Torsin1 in neurons, since loss-of-function phenotypes might not be mitigated by compensatory functions provided by closely related proteins. Similar to the relationship between human TorsinA and TorsinB, zebrafish Torsin 1a and Torsin 1b are most divergent within the N-terminal region. It is possible that discrete functions of TorsinA and TorsinB are distributed between the two isoforms encoded by the *tor1* gene in zebrafish. Compatible with this idea, the expression pattern of *tor1* showed similarities to both TOR1A and TOR1B. The relatively weak expression of both *tor1_tv1* and *tor1_tv2* in the brain, compared to gut and gonads resembles the expression of mammalian TOR1B, which is more abundantly expressed in somatic cells than neurons. Conversely, Torsin1-related immunoreactivity in the zebrafish nervous system appeared to closely resemble the expression pattern of TorsinA in the mammalian nervous system, with prominent localization in the cell body and processes of neurons and absence from white matter tracts. The development of isoform-specific antibodies to zebrafish Torsin1a and Torsin1b will allow clarification of whether the expression patterns of these two proteins are complementary and similar to mammalian TorsinA and TorsinB.

In order to characterize the zebrafish Torsin1 protein, we developed a novel antibody directed towards its C-terminus. We showed that: (i) the antibody recognizes a protein produced by expression of cDNA encoding *tor1* in cultured cells; and (ii) pre-incubation of the antibody with its cognate peptide prevented it from detecting epitopes on either western blot or tissue sections. These controls support the conclusion that the antibody specifically detected Torsin1 in zebrafish brain. However, loss of immunoreactive signal in genetic knockout tissue is generally considered a ‘gold standard’ for proving antibody specificity [63]; in this case, the absence of clearly demonstrable Torsin1 immunoreactivity during early development precluded confirmation of specificity by MO-mediated knockdown of the *tor1* transcript. Formal proof that the antibody specifically detects Torsin1 in the zebrafish brain therefore awaits the development of stable *tor1* ^−/−^ lines. Nonetheless, the availability of this antibody allowed some initial conclusions to be drawn about the nature of Torsin1: it is expressed as two species in CNS, a major 45 kDa form and a minor 47 kDa form; both species are heavily N-glycosylated, showing loss of 9 kDa carbohydrate after deglycosylation; and the protein localizes to neuronal somatic cytoplasm and possibly also nerve terminals within the neuropil, but was not detected within axons. These properties are similar to those previously reported for human TorsinA, and are compatible with an endoplasmic reticulum subcellular localization in neurons. The origin of the two protein species is unclear; the predicted molecular weights of Torsin1a and Torsin1b are similar (38.2 kDa and 38.4 kDa, respectively) and approximately the size of the major protein isoform found after deglycosylation. It is possible that different post-translational modifications are responsible for the two forms, or that signal peptide cleavage is directed to a different site in the nascent protein by each of the unique N-termini.

Relocalization of TorsinA from the endoplasmic reticulum to the nuclear envelope is a hallmark of the dystonia-associated ΔE mutation [12]. This relocalization can also be induced by mutations in the ATP-hydrolysis domain of TorsinA [9], [12]. Although we were not able to introduce the ΔE mutation into Torsin1, because of limited sequence conservation with respect to the human protein in this region of the C-terminus, we have demonstrated that zebrafish Torsin1 can be made to accumulate in the nuclear envelope by the introduction of a putative ATP-hydrolysis null mutation in the Walker B domain. Interestingly, this relocalization was observed in a heterologous murine cell line. Trafficking of Torsins between nuclear envelope and endoplasmic reticulum is thought to depend on their interactions with specific substrates and with other Torsin molecules in hexameric complexes. This raises the possibilities that the zebrafish protein may interact with: (i) one or more mammalian nuclear envelope proteins, possibly some of the same proteins that interact with endogenous mammalian Torsins; or (ii) endogenous mammalian TorsinA to form complexes. These possibilities are not mutually exclusive and both are equally compatible with the data presented. For example, even if zebrafish Torsin1 did not recognize murine nuclear envelope substrates, disruption of its ATPase domain may have prevented a Torsin1/TorsinA heterohexamer from disengaging from a substrate. These data show an intriguing degree of functional interaction between the heterologous proteins and imply that at least some of the cellular roles may be phylogenetically conserved. It will be of interest to determine whether human TorsinA behaves similarly in zebrafish cells, since this will have significant implications for generating a zebrafish model of DYT1 dystonia based on transgenic expression of human TorsinA[ΔE]. Zebrafish homologues of many of the human proteins known to interact with TorsinA have been described, and we have identified candidates for others by database searching (Table S1). It will be of considerable interest to determine whether these proteins interact with Torsin1, suggesting phylogenetic conservation of biochemical properties and functions.

We did not identify any morphological or neurobehavioral abnormalities resulting from reduced Torsin1 expression early in development, although the use of RNA in situ hybridization to label dopamine neurons does not allow examination of neuronal morphology and so the data do not exclude, for example, abnormalities of dendritic branching. The absence of prominent phenotypes in morphant larvae could be attributable to incomplete knockdown of Torsin1 (figure 10A, C) or compensatory functions provided by other Torsin proteins. However, these data are also consistent with findings in murine TOR1A*^−/−^* lines, in which the first observed phenotypic abnormality was perinatal lethality due to lack of feeding [22]. In zebrafish, the analogous developmental point occurs after 5 dpf, when patterning of the nervous system is nearly complete and larvae begin actively pursuing food. Unfortunately, by this time point, the expression of both *tor1* transcripts had largely recovered from transient MO-mediated knockdown (figure 10A, C); consequently it remains unclear whether loss of *tor1* in zebrafish at later developmental points would provoke similar abnormalities to those observed in mice lacking TorsinA. DYT1 dystonia patients usually first show symptoms during late childhood or adolescence. Consequently, it is expected that critical CNS functions of TorsinA, of relevance to the pathogenesis of dystonia, could occur much later during development. In order to address these possibilities and to elucidate the role of Torsin1 during later development, it will be necessary to develop stable *tor1* ^−/−^ lines. This would not only allow unambiguous evaluation of Torsin1 functions at later time points, but will also facilitate validation of Torsin1 antibodies for further biochemical studies.

In conclusion, our data suggest that the zebrafish may provide a powerful model system to study the neuronal functions of Torsin1 *in vivo*, and an opportunity to elucidate the molecular evolution of Torsins. Our work provides essential background for future studies on *tor1* gene function and raises the intriguing possibility of generating a transgenic zebrafish model of DYT1 dystonia, with potential applications in understanding pathogenesis and developing novel treatments.

## Supporting Information

Table S1 Zebrafish homologs of TorsinA-interacting proteins. Human proteins known to interact with TorsinA are shown in the first column and details of their genes shown in the second column. The third column shows details of the proposed zebrafish homologues of these genes (references are shown where available; otherwise, Sager et al this publication). The final column shows the degree of homology between the human and zebrafish proteins as % amino acid residues that are identical or homologous between human and zebrafish.
(PDF)
