# Supplementary material for: The Zebrafish Homologue of the Human DYT1 Dystonia Gene Is Widely Expressed in CNS Neurons but Non-Essential for Early Motor System Development
Source: PLoS One. 2012 Sep 28;7(9):e45175. doi: 10.1371/journal.pone.0045175 (PMC3460957; doi:10.1371/journal.pone.0045175)
Supplement: Table S1 — Zebrafish homologs of TorsinA-interacting proteins. Human proteins known to interact with TorsinA are shown in the first column and details of their genes shown in the second column. The third column shows details of the proposed zebrafish homologues of these genes (references are shown where available; otherwise, Sager et al this publication). The final column shows the degree of homology between the human and zebrafish proteins as % amino acid residues that are identical or homologous between human and zebrafish. (PDF) [file pone.0045175.s001.pdf]

Supplementary table 1: Zebrafish homologs of TorsinA-interacting proteins

Human proteins known to interact with TorsinA are shown in the first column and details of their genes shown in the second column. The third column shows details of the proposed zebrafish homologues of these genes (references where available are shown; otherwise, Sager et al this publication). The final column shows the degree of homology between the human and zebrafish proteins as % amino acid residues that are identical or homologous between human and zebrafish.

TorsinA interacting Protein	Human Gene	Zebrafish Homolog	Protein Homology (% identity/% homologous)
**Kinesin Light Chain 1** [1]	*KLC1*<br>Gene ID: 3831<br>Locus: 14q32.3<br>Protein Length: 560-618	*klc1a*<br>Gene ID: 393928<br>Chromosome: 13<br>Protein length: 538	71/77
		klc1b<br>Gene ID: 335499<br>Chromosome: 13<br>Protein length: 631	74/82
**Lamina-associated protein 1 (LAP1**) [2]	*TOR1AIP1*<br>Gene ID: 26092<br>Locus: 1q24.2<br>Protein Length: 583-584	*zgc:112962*<br>Gene ID: 548348<br>Chromosome: 8<br>Protein length: 324<br>**or**	LAP1 compared with:<br>*zgc:112962* 24/33<br>*si:dkeyp-82a1.6* 25/35
**Lumenal domain like LAP1 (LULL1)** [2]	*TOR1AIP2*<br>Gene ID: 163590<br>Locus: 1q25.2<br>Protein Length: 131, 470	*si:dkeyp-82a1.6*<br>Gene ID: 100005391<br>Chromosome: 8<br>Protein length: 548<br>(predicted gene only)	LULL1 compared with:<br>*zgc:112962* 19/26<br>*si:dkeyp-82a1.6* 21/31
**Snapin** [3]	*SNAPIN*<br>Gene ID: 23557<br>Locus: 1q21.3<br>Protein Length: 136	*snapin*<br>Gene ID: 567959<br>Chromosome: 19<br>Protein length: 129	72/79
**Tau** [4]	*MAPT*<br>Gene ID: 4137<br>Locus: 17q21.1<br>Protein Length: 381-776	*mapta* [5]<br>Gene ID: 567833<br>Chromosome: 3<br>Protein length: 784	33/43
		maptb [5]<br>Gene ID: 100000342<br>Chromosome: 12<br>Protein length: 337	41/51
**Vimentin** [6]	*VIM*<br>Gene ID: 7431<br>Locus: 10p13<br>Protein Length: 466	*vim (1 of 2)*<br>Gene ID: 140599<br>Chromosome: 24<br>Protein length: 455	69/85
		vim (2 of 2)<br>Gene ID: 393746<br>Chromosome: 2<br>Protein length: 447	66/82

**Nesprin-3** [7]	*SYNE3*<br>Gene ID: 161176<br>Locus: 14q32.13<br>Protein Length: 975	*zgc:158327* [8]<br>Gene ID: 100009645<br>Chromosome: 17<br>Protein length: 1093 or 1100	36/49
**Printor** [9]	*KLHL14*<br>Gene ID: 57565<br>Locus: 18q12.1<br>Protein Length: 628	*klhl14*<br>Gene ID: 794722<br>Chromosome: 24<br>Protein length: 607	88/92
**Tyrosine Hydroxylase** [10]	*TH*<br>Gene ID: 7054<br>Locus: 11p15.5<br>Protein Length: 497- 528	*th1* [11]<br>Gene ID: 30384<br>Chromosome: 25<br>Protein length: 489<br><br>*th2* [11]<br>Gene ID: 414844<br>Chromosome: 4<br>Protein length: 471	68/80<br><br><br><br><br>58/70
**CSN4** [12]	*COPS4*<br>Gene ID: 51138<br>Locus: 4q21.22<br>Protein Length: 352-406	*cops4*<br>Gene ID: 325592<br>Chromosome: 10<br>Protein length: 406	95/98
**Stonin 2** [12]	*STON2*<br>Gene ID: 85439<br>Locus: 14q31.1<br>Protein Length: 905-920	*zgc:113338*<br>Gene ID: 619262<br>Chromosome: 17<br>Protein length: 854	46/56

References:

1. Kamm C, Boston H, Hewett J, Wilbur J, Corey DP, et al. (2004) The early onset dystonia protein torsinA interacts with kinesin light chain 1. J Biol Chem 279: 19882-19892.
2. Goodchild RE, Dauer WT (2005) The AAA+ protein torsinA interacts with a conserved domain present in LAP1 and a novel ER protein. J Cell Biol 168: 855-862.
3. Granata A, Watson R, Collinson LM, Schiavo G, Warner TT (2008) The dystonia-associated protein torsinA modulates synaptic vesicle recycling. J Biol Chem 283: 7568-7579.
4. Ferrari-Toninelli G, Paccioretti S, Francisconi S, Uberti D, Memo M (2004) TorsinA negatively controls neurite outgrowth of SH-SY5Y human neuronal cell line. Brain Res 1012: 75-81.
5. Chen M, Martins RN, Lardelli M (2009) Complex splicing and neural expression of duplicated tau genes in zebrafish embryos. J Alzheimers Dis 18: 305-317.
6. Hewett JW, Zeng J, Niland BP, Bragg DC, Breakefield XO (2006) Dystonia-causing mutant torsinA inhibits cell adhesion and neurite extension through interference with cytoskeletal dynamics. Neurobiol Dis 22: 98-111.
7. Nery FC, Zeng J, Niland BP, Hewett J, Farley J, et al. (2008) TorsinA binds the KASH domain of nesprins and participates in linkage between nuclear envelope and cytoskeleton. J Cell Sci 121: 3476-3486.
8. Postel R, Ketema M, Kuikman I, de Pereda JM, Sonnenberg A (2011) Nesprin-3 augments peripheral nuclear localization of intermediate filaments in zebrafish. J Cell Sci 124: 755-764.
9. Giles LM, Li L, Chin LS (2009) Printor, a novel torsinA-interacting protein implicated in dystonia pathogenesis. J Biol Chem 284: 21765-21775.
10. O'Farrell CA, Martin KL, Hutton M, Delatycki MB, Cookson MR, et al. (2009) Mutant torsinA interacts with tyrosine hydroxylase in cultured cells. Neuroscience 164: 1127-1137.
11. Candy J, Collet C (2005) Two tyrosine hydroxylase genes in teleosts. Biochim Biophys Acta 1727: 35-44.
12. Granata A, Koo SJ, Haucke V, Schiavo G, Warner TT (2011) CSN complex controls the stability of selected synaptic proteins via a torsinA-dependent process. EMBO J 30: 181-193.
